# Engagement in and correlates of total cutaneous exams and skin self-exams among young melanoma survivors and their family

**DOI:** 10.1007/s10865-025-00589-4

**Published:** 2025-07-20

**Authors:** Sharon L. Manne, Deborah A. Kashy, Sherry Pagoto, Susan K. Peterson, Carolyn J. Heckman, Joseph Gallo, Adam Berger, David B. Buller, Alexandria Kulik, Sara Frederick, Morgan Pesanelli

**Affiliations:** 1 Section of Behavioral Sciences, Rutgers Cancer Institute of New Jersey, 120 Albany Street, Tower II, New Brunswick, NJ 08901 USA; 2 Department of Psychology, College of Social Science, Michigan State University, East Lansing, MI USA; 3 Department of Allied Health Sciences, College of Agriculture, Health, and Natural Resources, University of Connecticut, Storrs, CT USA; 4 Division of Cancer Prevention and Population Sciences, The University of Texas MD Anderson Cancer Center, Houston, TX USA; 5 Hackensack Meridian Jersey Shore Medical Center, Hackensack Meridian Health, Paramus, NJ USA; 6 Klein Buendel, Inc., Golden, CO USA; 7 OHRS Clinical, Rutgers Cancer Institute of New Jersey, New Brunswick, NJ USA; 8 Office of Academic Affairs, Rutgers School of Public Health, Piscataway, NJ USA

**Keywords:** Young adult survivors, Melanoma, Cancer risk, Skin self-examination, Clinical skin examination, Family risk

## Abstract

Young adult melanoma survivors and their close family (first degree relatives/FDRs) are at increased risk for developing a melanoma, but little is known about engagement in and correlates of their clinical skin exam (CSE) and skin self-examination (SSE) behaviors. Five hundred and seventy-four YA survivors and their FDRs completed an online survey assessing engagement in CSE and SSE, as well as measures of background factors, cognitive and psychosocial factors, CSE and SSE planning, and family influences. Approximately 90% of YAs had a CSE and 90% performed SSE in the last year, but engagement in CSE among FDRs was lower (63.2%, CSE; 64.9%, SSE). For CSE, females, a physician recommendation, fewer barriers, and more planning were associated with CSE. Family influences were not associated with CSE. For SSE, a physician recommendation and greater self-efficacy were associated with engagement, and more comprehensive SSE was associated with a physician recommendation, lower education, greater self-efficacy, and more planning. Stronger family normative influences were associated with more comprehensive SSEs among males. Findings suggest FDRs may benefit from interventions to improve CSE and SSE and indicate that physician recommendation may be a key intervention target to foster CSE and SSE.

**Supplementary Information:**

The online version contains supplementary material available at 10.1007/s10865-025-00589-4.

## Introduction

Young adults (YA) diagnosed with cancer, defined as individuals diagnosed between the ages of 18 and 39, have been identified by the National Cancer Institute as a growing population (Closing the Gap: Research and Care Imperatives for Adolescents and Young Adults with Cancer, 2006). Melanoma is the most lethal form of skin cancer and the third and fourth most common cancer diagnosed in YA females and males, respectively (Cancer Stat Facts: Cancer Among Adolescents and Young Adults (AYAs) (Ages 15–39)). Risks for recurrent or new cancers are also higher in the YA population: YA survivors are 9 times more likely to develop another melanoma than adults over 39 years of age (3). They are also 16 times more likely to develop a second malignancy than age-matched, non-cancer comparison groups (Chao et al., 2019) and have a higher relative risk for a second malignancy than adults diagnosed with cancer over 39 years of age (Lee et al., 2016). Further, although the 5-year relative survival for melanoma among YAs (97.6% for female, 93.3% for males) is higher than those for other cancers, a greater percentage of cases among YAs are diagnosed at later, less curable stages (22%) than adults over 39 years of age. (13%) (SEER*Stat Databases: November 2019 Submission, 2020).

Although the risk for recurrence or a new primary cancer is highest within the first five years following diagnosis for all melanoma survivors, survivors are at risk for a new primary melanoma or recurrent disease throughout their life (Brauer et al., 2010; Gerlini et al., 2018; Tsao et al., 1997; von Schuckmann et al., 2019). Regular follow-up surveillance for earlier detection is important for melanoma survivors, particularly among YAs, whose risk will likely span a longer period of time. Follow-up surveillance includes clinical skin exam (CSE) and thorough skin self-examination (SSE) which are recommended by professional groups such as the National Comprehensive Care Network and the American Cancer Society (Living as a melanoma skin cancer survivor, 2019; NCCN Clinical Practice Guidelines in Oncology: Melanoma; version 4.2011. Available at www.nccn.com.). Recommendations for regular SSE are supported by several lines of research regarding potential benefits. First, more than half of recurrences and new primary melanomas are detected by survivors themselves (Francken et al., 2007; McPherson et al., 2006; Moore Dalal et al., 2008). Second, individuals who perform SSE are diagnosed with significantly earlier-stage melanomas than those who do not perform it. Detection and treatment of recurrent disease and new primaries at earlier stages leads to improved survival, which is not accounted for by lead-time bias (Leiter & Garbe, 2008). Third, melanomas identified by SSE are thinner than those found incidentally (De Giorgi et al., 2015; Leiter & Garbe, 2008; McPherson et al., 2006; Pollitt et al., 2009). Retrospective studies suggest that individuals who perform SSE have lower tumor thickness, and thinner melanomas are associated with better survival (Lo et al., 2018; Murali et al., 2012; Pollitt et al., 2009; Schneider et al., 2008). Thus, promoting regular SSE will likely enhance early detection of easier to treat recurrences and new primaries among survivors.

In addition to the greater disease burden, research examining the survivorship experience among YAs indicate they encounter unique challenges, including managing survivorship care during a time of life when there are other life priorities, including pursuit of committed relationship, family, financial, and career goals (Barnett et al., 2016; Stone et al., 2017; Wong et al., 2017). Young adult survivors often face more psychosocial and financial challenges than other age groups (Lu et al., 2021). These unique challenges may interfere and compromise engagement in recommended surveillance and create higher needs for information about recommended follow-up care. (Barr et al., 2016; Grace et al., 2019; Keegan et al., 2012).

In this study, we adopted a family-focused approach to characterizing follow-up surveillance practices by including both survivors and their FDRs (siblings, parents, children). There were two reasons this approach was taken. First, FDRs have a two-fold risk for developing this cancer (Cho et al., 2005; Ford et al., 1995), and professional organizations recommend that FDRs engage in regular prevention and surveillance practices such as CSE and SSE (Bichakjian et al., 2011; NCCN Clinical Practice Guidelines in Oncology: Melanoma, 2016). Second, family influences may play a particularly strong role in engagement in risk-reducing behaviors in YA families given the unique challenges YA survivors and their family encounter. Among melanoma survivors of all ages, family influences are important. Family relationships may be even more salient among YA survivors, who often face disruptions and isolation from peer relationships (Warner et al., 2016). A melanoma diagnosis stimulates discussions among family members (Bowen et al., 2017; Loescher et al., 2009; Mujumdar et al., 2009). For families of survivors of other cancers, family discussions are associated with greater engagement in cancer screening (Mesher et al., 2014).

Unfortunately, despite the fact that YA melanoma survivors and their FDRs are at higher melanoma risk, few studies have focused specifically on adherence to surveillance practices such as CSE and SSE adherence, none have focused on FDRs, and none have included both survivor and FDRs. One study that included YAs in their sample and evaluated age differences (Reserva et al., 2017) found that younger survivors (< 50 years of age) were twice as likely to be non-adherent to CSE surveillance follow-up appointments than older survivors. Another study by Miller and colleagues (Miller et al., 2020) focused specifically on CSE and SSE among adolescent and YA melanoma survivors and found that two-thirds of their sample reported a CSE at least annually or more frequently. More than 80% reported SSE more recently than the last 6 months. There are no studies that have examined adherence to either CSE or SSE among FDRs of AYAs, and no studies have included both survivors and their FDRs.

Understanding factors associated with adherence to CSE and SSE among YA melanoma survivors and their FDRs would assist in designing more efficacious interventions. Unfortunately, the literature is very limited. Miller and colleagues (Miller et al., 2020) evaluated the role of demographic, medical, psychological, self-efficacy, and perceived risk in CSE and SSE adherence among adolescent and YA survivors. They found that higher SES, having a regular source of non-cancer care, and greater health care self-efficacy were associated with higher adherence to CSE, and Hispanic ethnicity was associated with lower adherence to CSE. Only greater self-efficacy was associated with higher SSE adherence. To date, there have been no studies evaluating factors associated with engagement in CSE and SSE among FDRs of YA survivors.

To address these gaps in the literature in this unique and growing population of YA cancer survivors and their families, the current study evaluated engagement in, and factors associated with, CSE and SSE among YA survivors and their FDRs. Our first aim was to examine rates of CSE and SSE engagement. The inclusion of both survivors and their family members allows us to evaluate not only levels of engagement, but also the ability to examine differences in engagement by family role (i.e., survivor, sibling, parent, child) and sex, and correspondence between family members’ behaviors (i.e., similarity in engagement). Understanding differential patterns of engagement, sex differences, and correspondence between family members will provide important information for interventions such as which family members and families are less adherent. We predicted that CSE and SSE rates would be higher among survivors than their FDRs. Based on prior research (Manne & Lessin, 2006), we proposed that females would be more likely to conduct SSE. The second aim was to examine factors associated with rates of engagement. Our selection of factors was guided by the Preventive Health Model (PHM) (Myers et al., 1994, 2007), an integrative framework drawn from the Health Belief Model (Becker, 1974; Janz & Becker, 1984; Rosenstock, 1974; Rosenstock et al., 1988) and the Theory of Planned Behavior (Ajzen, 1985, 1991). Constructs represented in the PHM include personal background factors, cognitive and psychosocial factors, behavioral intention factors, and social support and influence factors. In this study, background factors were examined were socio-demographics, objective skin cancer risk, medical history, and physician recommendations. The cognitive and psychosocial factors examined were perceived risk for melanoma, self-efficacy for performing CSE and SSE, CSE and SSE benefits, and CSE and SSE barriers. For behavioral intentions, we used Gollwitzer and colleagues’ construct of implementation intentions planning (Gollwitzer & Brandstätter, 1997; Orbell & Sheeran, 2000; Sniehotta et al., 2005), which are “if–then” plans that specify a future) situation and a planned response (If I encounter situation X, then I will respond with action Y”. Social factors focused on were family influences for CSE and SSE, which included family norms for CSE and SSE, perceived benefits for the family for engaging in skin cancer-risk reduction practices, and communication between family members about CSE and SSE. Empirical data on CSE and TCE among YA melanoma survivors and their FDRs is needed to inform the development of interventions for this unique but growing population.

## Methods

This study used data from an online baseline survey from the Young Melanoma Families clinical trial, designed to increase sun protection, SSE, and CSE among melanoma survivors and their FDRs using a family Facebook intervention. A description is available on the clinical trials.gov website (ClinicalTrials.gov ID NCT03677739). The study was approved by the Rutgers University IRB and other sites.

### Eligibility

Eligible survivors were/had: (1) diagnosed with stage 0–3 melanoma in the last 5 years; (2) aged 18 to 39 at diagnosis; (3) completed treatment at least 3 months previously; (4) no other concurrent cancer diagnosis; (5) able to speak and read English; (6) access to computer, internet, and a Facebook account; (7) at least one FDR consented to the study.

Eligible first degree relatives were/had: (1) age 18–80 years; (2) no personal history of melanoma; (3) full biological first degree relative (mother, father, sister, brother, son, daughter); (4) able to speak/read English; (5) access to computer, internet, and a Facebook account; (6) only one FDR with melanoma (the survivor); (7) not had a CSE in the past 3 years, had done SSE fewer than three times in the past year, or had an average item score on the Sun Protection Behavior index (Glanz et al., 2008) score < 4 (“often”) on a 5-point scale; (8) the survivor family member consented to the trial.

### Recruitment

Survivors were recruited from state registries and comprehensive cancer centers. For some registries, the registry confirmed eligibility, approached survivors, and provided contact information to the main study site. For other registries, the registry sent contact information to the main study site. For family expansion, survivors supplied contact information for their *full biological parents, any children over 18, and full biological siblings*. For cancer centers, recruitment and consent was conducted by that cancer center, and the main site staff handled data management afterwards. Survivors and FDRs were screened. If eligible, they were sent an electronic link to the consent. Families were enrolled when the survivor and at least one FDR consented. Participants acknowledged/accepted participation, *and they were paid $30 for completing the survey.*

2777 individuals were contacted. Of these, 367 could not be reached/contact information was incorrect, 451 were ineligible, 1366 refused, and 593 (281 survivors, 312 family) completed the survey (593/1959 of eligible/could be located; 30.1% response rate). Of the 281 survivors, 257 had a single family member participate, 17 had two members participate, and seven had three members participate. Family members included 150 mothers, 20 fathers, 95 sisters, 39 brothers, six daughters, and 3 sons. The small number of adult children made analyzing role differences in their data problematic, and these data were excluded. Dropping children left nine survivors without a FDR, and these survivors were dropped. The final sample of 574 participants was comprised of 272 survivors and 302 FDRs.

Comparisons between participants and refusers indicated that participants were diagnosed more recently [*t*(1315) = 2.37, *p* <.05, *M*_participants_ = 3.15, SD = 0.96, *M*_refusers_ = 3.4, SD = 1.73], were significantly younger [t(1318) = 3.8, *p* <.001, *M*_participants_ = 34.1, SD = 5.62, *M*_refusers_ = 35.43, SD = 5.32], and men were significantly more likely to refuse (Chi-square = 30.92, *p* <.001, Male_refusers_ = 77.8% Female_refusers_ = 64.7%).

### Measures

#### Background factors

*Demographics* Participants reported age, sex, race/ethnicity, education level, insurance status, and whether they see a primary care provider at least once a year. Phenotypic risk was calculated from eye color (*blue/green* = 1*, brown/hazel* = 0) and hair color (*blonde/red* = 1*, brown/black* = 0), with scores ranging from 0 to 2.

*Medical factors* Months since the survivors’ surgery, cancer stage, and cancer location were collected from survivor participants’ medical charts.

*Physician recommendation* Two items used in prior work (Manne & Lessin, 2006) assessed whether a doctor advised the participant to have CSE or conduct SSE (*yes/no*).

#### Cognitive and psychosocial factors

*CSE and SSE benefits* Nine items developed for melanoma survivors and their FDRs and used in prior work (Heckman et al., 2021; Manne & Lessin, 2006; Manne et al., 2011) assessed CSE benefits (Sample item: “Having a doctor examine my skin regularly will make me feel in control of my health”); 1 = strongly disagree, 5 = strongly agree; *α* = 0.89. Ten items developed for melanoma survivors and their FDRs and used in prior work (Heckman et al., 2021; Manne & Lessin, 2006; Manne et al., 2011) assessed SSE benefits (Sample item: “Regular skin self-checks would help me to live a long life”); 1 = strongly disagree, 5 = strongly agree; *α* = 0.91. For both scales, averages were computed, and higher scores indicate more benefits.

CSE and SSE barriers. Seven items developed for melanoma survivors and their FDRs and used in prior work (Heckman et al., 2021; Manne & Lessin, 2006; Manne et al., 2011, 2021) assessed CSE barriers (Sample item: “It would be embarrassing to have a doctor look at my entire body”); 1 = strongly disagree, 5 = strongly agree; *α* = 0.80. Ten items developed for melanoma survivors and their FDRs and used in prior work (Manne & Lessin, 2006; Manne et al., 2004) assessed SSE barriers (Sample item: “It would take too much time to do regular skin self-checks); 1 = strongly disagree, 5 = strongly agree; *α* = 0.80. For both scales, averages were computed, and higher scores indicate more barriers.

Perceived melanoma risk. An item adapted from Schwartz and colleagues (Zinman et al., 1995) and used in prior work (Manne & Lessin, 2006; Manne et al., 2004) assessed risk compared with others with the same melanoma family history (1 = much lower than other people, 5 = much higher than other people).

SSE self-efficacy. Eight items used in prior work (Coups et al., 2016; Manne & Lessin, 2006; Manne et al., 2004, 2021) assessed confidence in being able to conduct SSE (Sample item: “You know how to examine the skin on your body for signs of skin cancer”); 1 = not at all confident, 5 = very confident; *a* = 0.89. An average was computed, and higher scores indicate more efficacy.

CSE and SSE Action and Coping Planning. This scale was adapted for CSE and SSE from prior work using this construct (Luszczynska et al., 2007; Saddawi-Konefka et al., 2016; Schwarzer & Renner, 2000). Four items assessed whether participants made a detailed plan regarding when, where, and how to have a CSE and what to do if something interferes with plans (Sample item: I have made a detailed plan regarding when to have a physician skin exam); *a* = 0.95. Four items assessed whether participants made a detailed plan regarding when, where, and how to engage in SSE and what to do if something interferes with plans (Sample item: “I have made a detailed plan regarding how to engage in a skin self-check”); *a* = 0.96. For both scales, 1 = strongly disagree, 5 = strongly agree). An average was computed, and higher scores indicate more planning.

#### Family influence factors

Family norms for CSE and SSE (Scale name: Norms) Five items developed specifically for this study and based on assessed attitudes and practices of family members about CSE (Sample item: “My family members have had their skin examined by a physician”); *α* = 0.73. Six items assessed attitudes and practices of family members about SSE (Sample item: “My family thinks it is important for me to regularly examine all of my skin for growths or changes in spots and moles”); *α* = 0.81. For both scales, 1 = strongly disagree, 5 = strongly agree). An average was computed, and higher scores indicate stronger norms.

Family Benefits (Scale name: Family Perceptions). This eight nine item measure (Coups et al., 2018), used in our pilot study and adapted from our prior work (author details withheld for the review process), assessed perceived benefits to one’s family if the participant engages in skin cancer risk reduction practices as well as benefits for the individual if their family engages in these practices (Sample items: “If I engage in regular skin self-checks and physician skin examinations, it would be beneficial to my family,” “I can think of reasons that engaging in skin self-checks and physician skin examinations would be beneficial to my family members.”); 1 = strongly disagree, 5 = Strongly agree, not at all confident, 5 = very confident; α = 0.81. An average was computed, and higher scores indicate more perceived family benefits.

*Family discussion (scale name: discussion about screening* This measure was used in our pilot study (author details withheld for the review process) and adapted from our prior work (author details withheld for the review process). Three items assessed the frequency of discussions about CSE with siblings, parents, and spouse, and three items assessed the frequency of discussions about SSE with siblings, parents, and spouse (Sample item: “How often did you discuss skin self-checks in the past month with your spouse/partner); never = 0, once = 1, twice = 2, more than two times = 3. Because participants may not have one or more of these FDRs, the family discussion variable was computed by dividing the sum of the frequency of discussions by the number of available family member types and then multiplying by 100. A score of 100 indicates the person discussed CSE (or SSE) at least two times with available FDRs (CSE, *M* = 23.94, *SD* = 24.9; SSE, *M* = 21.57, *SD* = 25.4).^1^

#### Outcomes

*CSE* CSE was defined as a doctor deliberately checking any part of the participant’s body for early signs of skin cancer**.** Participants reported the last time a doctor did this (within the last month, 1–6 months ago, 7 to 12 months ago, more than 1 year ago, never). The outcome was assessed as whether the participant had CSE in the last year (yes/no).

*SSE* SSE was defined as any time the participant checked any part of their body for signs of skin cancer. To assess this, participants reported whether checked any part of their body for early signs of skin cancer in the last 12 months Thorough SSE was defined as spending time looking at the skin systematically and deliberately. Thorough SSE was assessed by asking participants who checked their skin at least once to indicated whether they thoroughly checked 15 body parts during their last SSE. Two outcomes were used: SSE performance in the past year: (yes/no) and SSE comprehensiveness (the number of body parts checked in the last SSE).

### Statistical methods

To address Aim 1, initial analyses predicting whether a person had completed a CSE/SSE used binary logistic multilevel models to account for non-independence due to family membership. Fixed effect predictors included role (survivor, parent, or sibling), sex, and the sex by role interaction. A random intercept for families was used to compute the intraclass correlation, which measures the correspondence within families in completing CSE/SSE. A standard multilevel analysis was conducted to predict SSE comprehensiveness using the same fixed and random effect parameters. To address Aim 2, we used a parallel multilevel approach with the same random intercept specification, but with different fixed effect predictors. A hierarchical approach tested whether: (1) adding demographic/medical variables improved model fit over and above sex and role; (2) adding individual attitudes improved model fit over and above the demographic/medical variables, and; (3) adding family attitudes and communication improved fit. Deviance tests evaluated incremental fit. At each stage, we tested for interactions involving sex and role.

## Results

### Descriptive statistics

FDRs were categorized as either parents or siblings and by sex. Descriptive data are shown in Table 1. Participants were primarily non-Hispanic white, were well-educated.

**Table 1:** Sample characteristics

	Survivor (n = 272)				Parent (n = 170)				Sibling (n = 132)				Total Sample (n = 574)			
	Male (n = 63)		Female (n = 209)		Father (n = 20)		Mother (n = 150)		Brother (n = 39)		Sister (n = 93)		Male (n = 122)		Female (n = 452)	
Variable	N (%)	M (SD)	N (%)	M (SD)	N (%)	M (SD)	N (%)	M (SD)	N (%)	M (SD)	N (%)	M (SD)	N (%)	M (SD)	N (%)	M (SD)
Age (years)		34.16 (7.23)		34.64 (5.59)		65.38 (5.62)		62.33 (6.90)		33.28 (6.93)		35.38 (8.11)		38.78 (13.41)		43.41 (14.34)
Race/Ethnicity																
Non-Hispanic White	61 (96.8)		199 (95.2)		20 (100.0)		141 (94.0)		37 (94.9)		87 (93.5)		118 (96.7)		427 (94.5)	
Non-Hispanic Black	0 (0)		0 (0)		0 (0)		0 (0)		0 (0)		0 (0)		0 (0)		0 (0)	
Non-Hispanic Asian	0 (0)		0 (0)		0 (0)		1 (0.7)		0 (0)		1 (1.1)		0 (0)		2 (.4)	
Hispanic	1 (1.6)		8 (3.8)		0 (0)		6 (3.9)		2 (5.1)		3 (3.2)		3 (2.5)		17 (3.8)	
American Indian/Alaska Native	0 (0)		1 (0.5)		0 (0)		1 (0.7)		0 (0)		1 (1.1		0 (0)		3 (0.7)	
Missing	0 (0)		1 (.5)		0 (0)		0 (0)		0 (0)		0 (0)		0 (0)		1 (0.2)	
Other	1 (1.6)		0 (0)		0 (0)		1 (0.7)		0 (0)		1 (1.1)		1 (.8)		2 (.4)	
Education																
< High school	0 (0)		0 (0)		0 (0)		1 (0.7)		0 (0)		0 (0)		0 (0)		1 (0.2)	
High school	2 (3.2)		10 (4.8)		0 (0)		18 (12.0)		1 (2.5)		2 (2.2)		3 (2.4)		30 (6.6)	
Some college	11 (17.5)		27 (12.9)		7 (35.0)		40 (26.7)		5 (12.8)		11 (11.8)		23 (18.9)		78 (17.3)	
Bachelor’s degree	29 (46.0)		87 (41.6)		7 (35.0)		44 (29.2)		18 (46.2)		49 (52.7)		54 (44.3)		180 (39.9)	
Graduate degree	21 (33.3)		85 (40.7)		6 (30.0)		46 (30.7)		15 (38.5)		31 (33.3)		42 (34.4)		162 (35.8)	
Missing	0 (0)		0 (0)		0 (0)		1 (0.7)		0 (0)		0 (0)		0 (0)		1 (0.2)	
Insurance (yes)	62 (98.4)		201 (96.2)		19 (95%)		144 (96%)		38 (97.4)		91 (97.8)		119 (97.5)		436 (96.5)	
Annual primary care visit (yes)	47 (74.6)		185 (88.5)		17 (85.0)		140 (94.0)		24 (61.5)		77 (82.8)		88 (72.1)		490 (85.5)	
Phenotypic risk																
0	21 (33.3)		61 (29.3)		6 (30)		59 (39.3)		12 (30.8)		41 (44.1)		39 (32.2)		161 (35.8)	
1	32 (50.8)		71 (34.1)		10 (50)		48 (32)		21 (53.8)		29 (31.2)		62 (51.2)		147 (32.7)	
2	10 (15.9)		76 (36.5)		4 (20)		43 (28.7)		6 (15.4)		23 (24.7)		20 (16.5)		142 (31.6)	
Time since melanoma diagnosis (months)		3.21 (0.86)		3.17 (0.99)												
Disease stage																
0	16 (25.4)		56 (26.8)													
1	25 (39.6)		93 (44.5)													
2	5 (7.9)		12 (5.7)													
3	12 (19.0)		19 (9.1)													
Missing	7 (11.0)		29 (13.9)													
Disease location																
Head or neck	16 (25.8)		16 (6.0)													
Trunk	29 (46.7)		85 (32.0)													
Extremity	17 (27.4)		103 (38.7)													

#### Aim 1: sex and role differences in CSE and SSE behaviors and family correspondence

Table 2 presents the CSE and SSE engagement for survivors and FDRs (siblings and parents together). Among survivors, 90.1% had a CSE in the last year and 89.6% performed a SSE in the last year. Among FDRs, 63.2% reported a CSE in the last year and 64.9% performed SSE in the last year. Table 3 presents CSE and SSE engagement as a function of family role, sex, and the role by sex interaction. The intraclass correlations that estimate within family correspondence in these behaviors are in Table 3. Intraclass correlations indicated little evidence of similarity for CSE or SSE within families: ICCs were small (all ICCs <.12), and none attained statistical significance.

**Table 2:** Engagement in CSE and SSE for survivors and first degree relatives

Variable	Survivors (N = 272)		Family members (n = 302)	
	N (%)	M (SD)	N (%)	M (SD)
CSE	245 (90.1)		191 (63.2)	
SSE (yes/no)	241 (89.6)		194 (64.9)	
SSE comprehensiveness		11.34 (3.29)		10.39 (3.63)
Body parts examined thoroughly in the last SSE				
Scalp	61 (25.5)		59 (30.4)	
Face	232 (96.3)		183 (93.8)	
Neck	215 (89.2)		164 (84.1)	
Shoulders	211 (89.7)		162 (83.1)	
Front of arms	225 (93.4)		182 (93.3)	
Back of arms	202 (83.8)		147 (75.4)	
Chest	229 (95.0)		178 (91.3)	
Stomach	223 (92.5)		167 (85.6)	
Upper back	163 (68.2)		119 (61.3)	
Lower back	156 (65.5)		120 (61.5)	
Front of legs	220 (91.3)		177 (58.6)	
Back of legs	182 (75.5)		143 (73.0)	
Bottom of feet	129 (54.0)		72 (36.9)	
Buttocks	145 (60.4)		89 (45.5)	
Genitals	141 (59.0)		74 (38.9)	
Examined all the above body parts	35 (12.9)		18 (5.9)

**Table 3:** Regression analyses predicting engagement in CSE, SSE (yes/no) and SSE comprehensiveness as a function of respondent role (survivors, parents, and siblings), respondent sex, and their interaction

		Survivor		Parent		Sibling		Sex main effect means		*F*_*sex*_ (*df*)	*F*_*role*_ (*df*)	*F*_role__*__sex_ (*df*)	ICC
		*M*	SE	*M*	SE	*M*	SE	*M*	SE				
CSE	Female	.90	.02	.44	.04	.36	.05	.62	.03	5.88*	55.70**	1.41	.046
	Male	.89	.04	.31	.10	.13	.05	.45	.07	(568)	(568)	(568)	
Role main effect means		.90	.02	.37	.06	.22	.05						
SSE	Female	.92	.02	.68	.04	.68	.05	.79	.02	7.97**	19.46**	0.14	.115
	Male	.82	.05	.51	.11	.54	.08	.64	.05	(562)	(562)	(562)	
Role main effect means		.88	.02	.60	.06	.61	.05						
SSE comprehensive	Female	11.35	.25	10.87	.34	10.06	.43	10.56	.47	1.63	5.31**	0.91	.099
	Male	11.34	.48	9.18	1.08	9.80	.73	10.10	.21	(428)	(364)	(413)	
Role main effect means		11.34	.27	10.02	.57	9.93	.43						

Tabled values are estimated marginal means (M) and standard errors (SE). F-Tests for CSE and SSE were conducted using binary logistic multilevel models and F-Tests for SSE Comprehensiveness were conducted using standard multilevel models. *df* = denominator degrees of freedom. Numerator *df* for role and the role by sex interaction is 2 and numerator *df* for sex is 1

*p <.05; **p <.01

There were significant main effects for sex and role, but no interaction predicting CSE. Across roles, females were more likely to have CSE in the last year: 62% of females had a CSE and 44% of males had a CSE. Survivors were most likely to have had a CSE (90%), followed by parents (37%) and siblings (22%). Post-hoc Bonferroni comparisons indicated that CSE engagement for survivors differed significantly from parents and siblings, but parents and siblings did not differ from one another.

Results for SSE indicated sex and role had significant main effects, but there was no interaction. Females were more likely to report SSE, and survivors were more likely to report SSE than parents and siblings. Post-hoc tests indicated that survivors differed significantly from parents and siblings, but parents and siblings did not differ from each other. Table 2 presents the number of participants who checked each body part. Table 3 presents the multilevel modeling results for the sex by role analysis on the number of body parts checked. To avoid overlap with the dichotomous SSE variable, for comprehensive SSE, only participants who reported examining at least one body part were included. For this variable, the only significant effect was the main effect of role. Survivors reported examining 11.34 body parts, parents reported examining 10.02 body parts, and siblings reported examining 9.93 body parts. Post-hoc tests indicated that survivors examined significantly more areas of their bodies than siblings, but parents did not differ from survivors or siblings. For both survivors and FDRs (see Table 2), the least commonly-reported areas checked were the scalp, bottom of feet, buttocks, and genitals.

#### Aim 2: demographic, medical, attitudinal, and family influences of CSE and SSE

Among demographic factors, multilevel regression models included only education, doctor recommendation, and phenotypic risk. Other demographic variables not included were age, because it covaried with family role, and race/ethnicity, because 96% were non-Hispanic White. Medical variables not included were: insurance (97% insured) and primary care provider visits (85% yearly visits). Stage was not included because a lack of data from a cancer registry resulted in many missing values (approximately 14% of survivors). The model predicting CSE (Table 4), included main effects for sex and role, education, doctor recommendation, phenotypic risk, benefits, barriers, planning, perceived risk, family benefits, family discussion, and family norms.

**Table 4:** Multilevel logistic regression analysis results predicting Clinical Skin Examination

	Predicting CSE				
	b	se	*p*	Exp(b)	χ^2^
Education	.184	.302	.541	1.202	
Doctor recommends CSE	1.655	.448	.000	5.232	
Phenotypic risk	.153	.164	.353	1.165	
					119.20**
CSE benefits	.171	.386	.658	1.187	
CSE barriers	−.657	.220	.003	.518	
CSE planning	.752	.171	.000	2.122	
Perceived risk	−.292	.245	.235	.747	
					121.09**
Family benefits	.221	.289	.445	1.247	
Family discussion of CSE	.004	.006	.495	1.004	
Family norms for CSE	.243	.180	.176	1.276	
					1.10

Education is coded 1 = a bachelor’s degree or higher, 0 = less than a bachelor’s degree. Doctor recommendation is coded 1 = yes, 0 = no. *Although not shown, all models included sex and role (survivor, parent, sibling) as predictors*. Chi-square tests indicate whether adding the preceding variables significantly improved model fit. All tabled coefficients are from the final model that included all 12 predictors

**p <.001

Controlling for other variables, individuals reporting a doctor recommendation were 5.32 times more likely to report CSE compared to individuals without. The chi-square test indicated that education, doctor recommendation, and phenotypic risk significantly improved model fit over and above a model that only included sex and role. For individual attitudes, higher barriers were associated with lower CSE, and more planning was associated with higher CSE. The final model included family influence factors, but none predicted CSE. Including the three variables together in the model did not significantly improve model fit.

Analyses predicting SSE and comprehensiveness are in Table 5. Controlling for other variables, participants with a doctor recommendation were 3.03 times more likely to complete SSE. For SSE comprehensiveness, participants with a doctor recommendation examined 1.24 more body parts than those without. Less educated participants examined.94 more body parts. Self-efficacy predicted SSE, and both self-efficacy and planning predicted more comprehensive SSE. Higher family benefits predicted a higher likelihood of SSE, but the association between benefits and comprehensiveness was negative. For both outcomes, including family variables improved model fit.

**Table 5:** Multilevel logistic regression analysis results predicting SSE (Yes/No) and multilevel regression results predicting SSE comprehensiveness (number of body parts)

	Predicting SSE (yes/no)					Predicting SSE comprehensiveness			
	b	se	*p*	Exp(b)	χ^2^	b	se	*p*	χ^2^
Education	.202	.299	.500	1.224		−.941	.346	.007	
Doctor recommends SSE	1.109	.318	.001	3.030		1.244	.534	.020	
Phenotypic risk	−.214	.166	.198	.807		.308	.175	.079	
					42.80**				51.81**
SSE benefits	−.132	.262	.616	.877		.435	.375	.247	
SSE barriers	.008	.241	.973	1.008		−.283	.283	.319	
SSE planning	.176	.128	.168	1.193		.802	.159	.000	
Perceived risk	.250	.222	.260	1.284		−.185	.282	.512	
SSE self-efficacy	.553	.184	.003	1.738		1.018	.212	.000	
					93.95**				141.72**
Family benefits	.975	.309	.002	2.651		−.901	.454	.048	
Family discussion of SSE	.001	.007	.887	1.001		.008	.006	.197	
Family norms for SSE	.274	.179	.126	1.315		.329	.207	.114	
					63.66**			20.46**	

Education is coded 1 = a bachelor’s degree or higher, 0 = less than a bachelor’s degree. Doctor recommendation is coded 1 = yes, 0 = no*. Although not shown, all models included sex and role (survivor, parent, sibling) as predictors.* Chi-square tests indicate whether adding the preceding variables significantly improved model fit. All tabled coefficients are from the final model that included all 13 predictors

**p <.001

Two interactions emerged from the analyses of predictors and sex. There was a significant interaction between family discussion and sex, F (1,537) = 6.81, *p* =.009. The association between family discussion and SSE was higher, but not statistically significant, for males (b =.038, se =.020, *p* =.054), but not for females (b =.003, se =.007, *p* =.688). There was a significant interaction between sex and family norms predicting comprehensiveness, F (1429) = 5.88, *p* =.016. For each unit increase in family norms, males reported examining 1.218 (se =.420, *p* =.004) additional body parts. There was no association between family norms and comprehensiveness for females (b =.088, se =.228, *p* =.702).

## Discussion

YA melanoma survivors and their FDRs are a growing population who are at elevated risk, but little is known about their engagement in CSE and SSE or the role of demographic, medical, cognitive and psychosocial, and family influences in CSE and SSE.

We will discuss the findings regarding engagement in CSE first, followed by SSE.

In terms of CSE, the fact that 90% of YA survivors had CSE in the last year was high and similar to engagement levels reported by Bowen and colleagues (Bowen et al., 2012) in their study of survivors of all ages. However, rates of CSE (63%) were lower among FDRs than YA survivors. FDRs of YA melanoma survivors were more likely to have had CSE as compared with prior studies targeting FDRs of all ages (55% (Manne et al., 2004); 47% (Azzarello et al., 2006)). When considered separately by family role, siblings’ CSE rates were similar to prior work focusing on siblings of survivors of all ages (27%) (Geller et al., 2003). Unfortunately, prior studies that include parents (Azzarello & Jacobsen, 2007; Manne et al., 2004) did not report uptake separately for different family roles, making direct comparisons difficult. We confirmed the expected sex differences found in prior work (Manne & Lessin, 2006; Robinson et al., 1998, 2002; Weinstock et al., 1999). CSE was much more common among females than males. Our findings extend prior work by illustrating that female FDRs are also more likely to have CSE. In our prior work among FDRs of all ages, sex was not associated with CSE (author details withheld for the review process). Correspondence between survivors’ and FDRs’ CSE was not significant, which was unexpected.

In terms of correlates of CSE, our findings underscore the importance of physician recommendation, barriers to CSE, and CSE planning. Physician recommendation has been a consistent correlate of CSE among survivors of all ages (Manne et al., 2004) and their FDRs (Azzarello & Jacobsen, 2007; Manne & Lessin, 2006). The association between fewer barriers and more CSE engagement is consistent with findings among survivors of all ages (Manne et al., 2004) and their FDRs (Azzarello & Jacobsen, 2007; Manne et al., 2015). Planning is an unstudied correlate, and our results support its role. Higher education and phenotypic risk were associated with a lower likelihood of CSE, consistent with work among FDRs of survivors of all ages (Azzarello et al., 2006; Geller et al., 2003; Manne et al., 2004). However, although education and phenotypic risk contributed to the overall regression model, neither was significant when they were included along with other factors. Finally, inconsistent with our predictions, family influences were not important.

Our findings suggest that SSE engagement was similar to studies of survivors of all ages (author details withheld for the review process) and their FDRs (62% (Geller et al., 2003); 71% (Manne et al., 2016)). Females were more likely to conduct SSE than men, which is consistent with studies of survivors and FDRs of all ages (Geller et al., 2003; Manne et al., 2004). Survivors were more likely to conduct SSE than their parents and siblings. It is impressive that the YA survivors and FDRs who conducted SSE engaged in relatively thorough self-examinations. It is also important to note that that SSE comprehensiveness among YA melanoma survivors was higher than rates documented in prior work [*M* = 7.6 (Coups et al., 2016)].

Correspondence between YA survivors’ and FDRs’ SSE was also not significant, which was unexpected. Physician recommendation was a strong correlate of SSE performance and comprehensiveness. As with prior work, self-efficacy predicted the likelihood of conducting SSE and SSE comprehensiveness (Coups et al., 2016). Planning predicted a more comprehensive SSE. Research focusing on survivors of all ages has suggested that perceived risk (Glenn et al., 2017; Robinson et al., 2002), benefits, and barriers are associated with this behavior (Manne & Lessin, 2006). It is possible that these factors are less important among YA survivors and their families, but further research is needed. Among family influence factors, only perceiving more family benefits was associated with SSE.

### Study limitations

One key limitation is that this is a cross-sectional study and causal inferences cannot be made. Second, although the percentage of FDRs who were ineligible due being adherent to recommendations was very low, exclusion may have biased our findings. Third, our 31% response rate was typical for a family expansion study, but not high, and may impact the generalizability. Participants were younger, diagnosed more recently, and more likely to be female than refusers. Future studies would benefit from stronger recruitment efforts targeted to males. Fourth, we did not assess whether participants lived together, which may increase family influences. Finally, our sample was comprised of primarily white non-Hispanic survivors (96.2%), and 76% had a college or higher education. Although the minority representation is slightly higher than published incidence rates for melanoma survivors in the US (Melanoma Incidence and Mortality, United States–2012–2016, 2019), future research should oversample minority survivors and include survivors and families who are less educated.

### Clinical implications and future research

Interventions seeking to increase CSE in YA melanoma survivors and their FDRs may be more effective if they focus on increasing physician recommendation, planning for CSE, and reducing perceived CSE barriers, rather than on enhancing family influences. Although our sample of male FDRs was small, targeting brothers and fathers in these efforts may be important due to their lower engagement. Our findings suggesting that YA melanoma survivors were less likely to engage in SSE are consistent with other studies of this population which have documented higher rates of adverse medical outcomes among male YA melanoma survivors and suggest that future studies may benefit from targeting male YA melanoma survivors (Gingrich et al., 2020). Interventions to increase SSE may benefit from fostering physician recommendations, improving SSE self-efficacy and planning, and pointing out benefits to one’s family for performing SSE. Although there was little family correspondence in SSE performance, fostering discussions and strengthening family norms about SSE may be useful intervention targets for male YA survivors, brothers, and fathers. Future research might benefit from efforts to increase participation among males as they were not well-represented in this sample. Finally, longitudinal data might provide a deeper understanding of how families of young survivors manage their risk-reduction practices, especially with a longer time since diagnosis or disease recurrence.

## Supplementary Information

Below is the link to the electronic supplementary material.

Supplementary file1 (DOCX 42 KB)

## Data Availability

De-identified data are available upon request. The data generated for this study are not publicly available due to our confidentiality commitment to our participants, but we will release de-identified data after a reasonable request is discussed with the IRB.

## Abbreviations

FDR: First degree relative
SSE: Skin self-examination
CSE: Clinical skin exam

## Footnotes

1 Although we know that the person discussed CSE and SSE with at least one sibling, we do not know if they discussed it with every sibling they have.
