# Supplementary material for: Engagement in and correlates of total cutaneous exams and skin self-exams among young melanoma survivors and their family
Source: J Behav Med. 2025 Jul 20;48(5):834–47. doi: 10.1007/s10865-025-00589-4 (PMC12474582; doi:10.1007/s10865-025-00589-4)
Supplement: Supplementary file 1 — Supplementary file1 (DOCX 42 KB) [file 10865_2025_589_MOESM1_ESM.docx]

**Not Statused** = 133

**Did not complete BL =** 81

**Dropped = 1**

Includes 1 patient who enrolled but asked to be removed from study

**In-limbo**= 10

Includes probands who consented or were eligible, or FDRs who consented or were eligible

**Enrolled = 607 - 43 = 574 total (excluding children and spouses)**

**Refused** = 1366

**Ineligible** = 436

Did not Consent: 4

Incorrect Phone Number/NIS: 363

Total Cases = 3001
